# A lightweight trichosanthes kirilowii maxim detection algorithm in complex mountain environments based on improved YOLOv7-tiny

**DOI:** 10.1371/journal.pone.0320315

**Published:** 2025-04-01

**Authors:** Zhongjian Xie, Xinwei Chen, Weilin Wu, Yao Xiao, Yuanhang Li, Yaya Zhang, ZhuXuan Wan, Weiqi Chen

**Affiliations:** 1 College of Physics and Electronic Information, Guangxi Minzu University, Nanning, China; 2 Guangxi Key Laboratory of Machine Vision and Intelligent Control, Wuzhou University, Wuzhou, China; UPES Dehradun, INDIA

## Abstract

Detecting *Trichosanthes Kirilowii* Maxim (Cucurbitaceae) in complex mountain environments is critical for developing automated harvesting systems. However, the environmental characteristics of brightness variation, inter-plant occlusion, and motion-induced blurring during harvesting operations, detection algorithms face excessive parameters and high computational intensity. Accordingly, this study proposes a lightweight *T.Kirilowii* detection algorithm for complex mountainous environments based on YOLOv7-tiny, named KPD-YOLOv7-GD. Firstly, improve the multi-scale feature layer and reduce the complexity of the model. Secondly, a lightweight convolutional module is introduced to replace the standard convolutions in the Efficient Long-range Aggregation Network (ELAN-A) module, and the channel pruning techniques are applied to further decrease the model’s complexity. Finally, the experiment significantly enhanced the efficiency of feature extraction and the detection accuracy of the model algorithm through the integration of the Dynamic Head (DyHead) module, the Content-Aware Re-Assembly of Features (CARAFE) module, and the incorporation of knowledge distillation techniques. The experimental results showed that the mean average precision of the improved network KPD-YOLOv7-GD reached 93.2%. Benchmarked against mainstream single-stage algorithms (YOLOv3-tiny, YOLOv5s, YOLOv6s, YOLOv7-tiny, and YOLOv8), KPD-YOLOv7-GD demonstrated mean average precision improvements of 4.8%, 0.6%, 3.0%, 0.6%, and 0.2% with corresponding model compression rates of 81.6%, 68.8%, 88.9%, 63.4%, and 27.4%, respectively. Compared with similar studies, KPD-YOLOv7-GD exhibits lower complexity and higher recognition speed accuracy, making it more suitable for resource-constrained *T.kirilowii* harvesting robots.

## 1 Introduction

*Trichosanthes kirilowii* Maxim (Cucurbitaceae), a perennial climbing herb, has garnered significant attention for its pharmacological properties, including expectorant, antibacterial, and antitumor activities [1]. Phytochemical studies identify its pericarp, seeds, and rhizomes as valuable sources of bioactive compounds, particularly seed-derived polysaccharides demonstrating dual immunomodulatory and antitumor effects with translational potential in pharmaceutical and biotechnological industries [2,3]. This species predominantly inhabits shaded slopes with forest understories and shrub vegetation at 200–1800 m elevations, distributed across Liaoning Province, northern China, and East Asia (e.g., Korea and Japan). However, current cultivation and harvesting remain dependent on labor-intensive manual practices, resulting in prolonged operational cycles and suboptimal yields that severely limit industrial scalability. To address these challenges, the development of automated high-precision identification systems is critical to standardize large-scale production and enable mechanized harvesting, thereby advancing the sustainable industrialization of *T.Kirilowii*.

Existing identification methods are divided into traditional machine vision and deep learning methods. Traditional machine vision detection algorithms are primarily based on color, shape, texture, etc. Xu et al. [4], designed the Directional Gradient Histogram algorithm based on HSV and a support vector machine classifier to detect strawberries under different environmental backgrounds, achieving a recognition rate of 87%. However, the precision of this approach diminishes in scenarios involving intricate image backgrounds. Arefi et al. [5] developed a new segmentation algorithm to extract tomato characteristics under greenhouse lighting conditions based on combining RGB, HSI, and YIQ color space. They guided the robotic arm to pick ripe tomatoes, achieving a total accuracy of 96.36% and a rapid response time of 2.2 seconds. However, this method exhibits obvious sensitivity to fluctuations in mountain lighting conditions. The above analysis highlights that traditional machine vision methods exhibit slower recognition speeds and depend on the environment. When the background changes in a complex way, the robustness and generalization ability decrease, posing a challenge to the practical application of harvesting robots in agricultural production.

In recent years, deep learning technology has rapidly developed and been widely applied in various fields, including medical diagnostics, autonomous driving, and intelligent agriculture. Object detection algorithms based on deep learning demonstrate high accuracy, speed, and generalization capabilities. Currently, there are primarily two types. One is two-stage object detection algorithms, such as R-CNN [6], Fast R-CNN [7], and Mask R-CNN [8]. Zhong et al. [9] combined an enhanced Faster R-CNN model with depth information to locate clustered peppers, achieving an average precision (AP) of 87.30%. Kumar [10] proposed a wheat leaf virus detection algorithm based on Mask R-CNN, achieving a detection accuracy of 97.16%. The other is single-stage object detection algorithms such as YOLO [11] and SSD [12]. Sekharamacntry et al. [13] introduced an enhanced real-time Apple detection model based on YOLOv5, achieving a detection accuracy of 97%. Lu et al. [14] proposed the Swin-transformer-YOLOv5 model for real-time grape cluster detection, with a maximum mean average precision of 97%. Single-stage object detection algorithms generally have slightly lower accuracy than two-stage object detection algorithms. However, they offer faster real-time detection speeds, making them more suitable for mountain agricultural product detection applications. Therefore, in this study, a single-stage detection algorithm was chosen for the real-time detection of *T.Kirilowii*.

In agricultural fruit object detection, researchers are dedicated to enhancing model detection accuracy, speed, and lightweight design to meet the demands of complex mountainous agricultural operations. Zhang et al. [15]. Introduced a lightweight Apple detection model based on YOLOv4 by incorporating lightweight network architectures such as GhostNet and depthwise separable convolution. This approach increased the average precision to 95.72% while compressing the model to 37.9M, representing only 15.53% of the size of the YOLOv4 weights. Shang et al. [16]. Designed a YOLOv5s lightweight model for apple blossom detection, utilizing ShuffleNetv2 and Ghost modules. The model size is only 0.61M, reducing memory usage and optimizing network structure. Concurrently, Miao et al. [17]. Introduced a lightweight YOLOv7 model for detecting cherry tomato ripeness. This approach reduced parameter volume by incorporating the MobileNetV3 module and enhanced accuracy by including a GAM attention mechanism, resulting in a 12% reduction in model size and a 0.1% increase in accuracy. The work above enhances the applicability of resource-constrained devices and provides feasible design strategies for lightweight agricultural fruit detection models. This is achieved by replacing lightweight structures or networks, reducing model parameter volume and complexity, and minimizing memory usage. Compared to non-lightweight detection methods, they are more suitable for real-time object detection in mountainous environments.

In summary, the object detection algorithm based on deep learning excels in extracting image features and is less affected by environmental factors. However, concerning the task of *T.Kirilowii* detection, YOLOv7-Tiny has some limitations, such as detection algorithms with lower accuracy and high computational complexity. Therefore, this study proposes a lightweight object detection method based on the improved YOLOV7-tiny to overcome the existing limitations. It aims to offer technical support for identifying and harvesting *T.Kirilowii* in complex mountainous environments. The contributions of this experiment are as follows:

(1) The multi-scale object detection layer has undergone optimization to screen out a model structure suitable for the detection of *T.Kirilowii*.
(2) The backbone network of the Efficient Long-range Aggregation Network (ELAN-A) has been restructured by incorporating Distributed Shifted Convolution (DSConv) and GSConv. Furthermore, the neck network has been optimized through the introduction of the Slim-neck lightweight network structure, with an additional application of channel pruning techniques to further decrease the complexity of the model.
(3) The experiment significantly enhanced the efficiency of feature extraction and the detection accuracy of the model algorithm through the integration of the Dynamic Head (DyHead) module, the Content-Aware Re-Assembly of Features (CARAFE) module, and the incorporation of knowledge distillation techniques.

The rest of the work is organized as follows. The second section provides a detailed introduction to the collection and processing of the datasets used in this experiment, an analysis of the YOLOv7-tiny algorithm, and the improvements made to the KPD-YOLOv7-GD algorithm. The third section presents the experimental results and their analysis, encompassing trials on improving multi-scale feature layers, lightweight experiments, experiments for enhancing lightweight accuracy, knowledge distillation experiments, ablation experiments, and comparative experiments with mainstream single-stage lightweight object detection algorithms. The fourth section discusses the advantages and limitations of the proposed improved model through visual analysis and comparative analysis of recognition performance among similar algorithms. Finally, the fifth section concludes the article.

## 2 Materials and methods

### 2.1 Image acquisition and preprocessing

**Fig 1:**
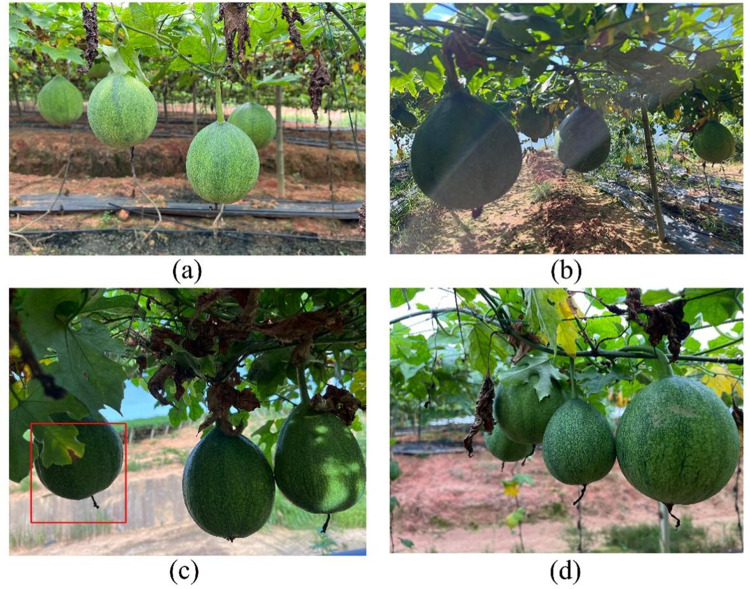
Images of *T.* *Kirilowii* under various lighting conditions in different mountainous terrains (a) normal light (b) backlight (c) leaf obstruction (d) clusters.

The *T.Kirilowii* images were collected from 1 July 2023 to 31 August 2023 in *T.Kirilowii* Industrial Base, Linchuan District, Fuzhou City, Jiangxi Province, China. As this area is an open research site, no special permits or authorizations were required for data collection. The image acquisition equipment utilized was the built-in camera of the DJI MM1A model Unmanned Aerial Vehicle (UAV). The drone’s flight distance was set to 0.3 to 0.5 meters to simulate real harvesting robot vision recognition scenarios. To accommodate varying natural lighting conditions across different mountainous terrains and ensure the algorithm’s adaptability to real operational scenarios, images of *T.Kirilowii* in various backgrounds were captured. The background conditions include normal light, backlight, leaf obstruction, and soil color. The dataset contains 1,600 images at a resolution of 3,968 ×  2,976 pixels. The images were resized to 640 × 640 pixels to facilitate model training and saved in JPG format. Fig.1 illustrates the *T.Kirilowii* under varying lighting conditions across mountainous terrains.

**Fig 2.**
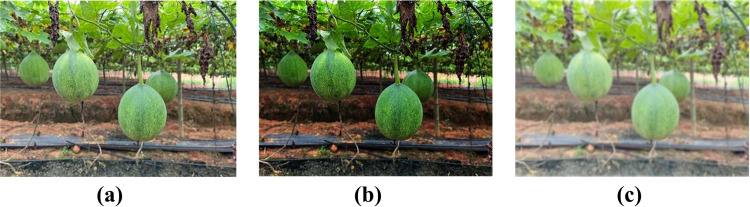
Data augmentation methods. (a) normal light (b) Random brightness; (c) Random Gaussian blurring.

To effectively address the impacts of varying light conditions and motion blur during the harvesting process of robots operating in mountainous environments, this study implemented a data augmentation strategy incorporating randomized brightness adjustment (0.5–1.5) and Gaussian blurring (kernel size: 3–11 pixels). The effect of data augmentation is shown in Fig.2. The total number of images after data augmentation amounts to 2,200. These images were randomly divided into training, validation, and test sets at a ratio of 8:1:1, respectively, ensuring no repetition within each group. This experiment obtained 1,760 training images, 220 validation images, and 220 test images. The distributions of the dataset are shown in Table 1.

**Table 1:** Quantitative description of the *T.Kirilowii* dataset.

Dataset	Original dataset (images)	Data augmentation (images)		Total (images)	Number of labels
		brightness variation	Gaussian blurring		
Train	1280	320	160	1760	44264
validation	160	40	20	220	5409
Test	160	40	20	220	55002
Total	1600	400	200	2200	55002

### 2.2 YOLOv7-tiny algorithm analysis

**Fig 3.**
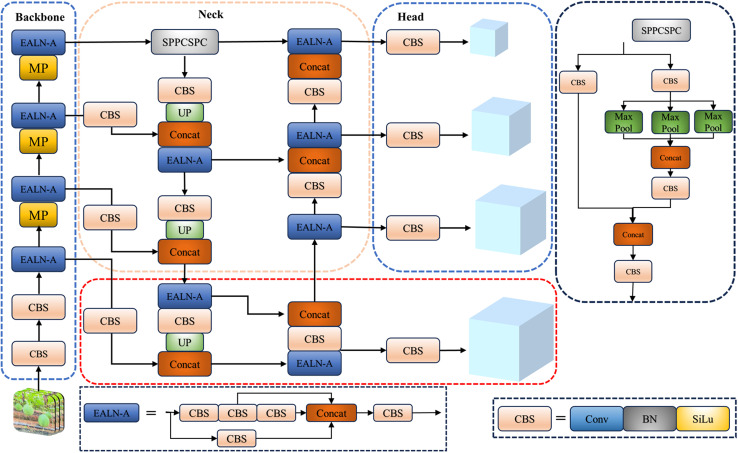
Structure of the YOLOv7-tiny network. The backbone network comprises layers of CBS convolution, ELAN-A, and MPConv convolution. Although the ELAN-A layer enhances the network’s feature extraction speed and simplifies the backbone structure of YOLOv7, the parameter and computational overhead remain considerable, making it unable to meet the demands of lightweight applications and small-scale devices.

The purpose of this experiment is to propose a lightweight model for detecting *T.Kirilowii*, and YOLOv7-tiny adopts this model because of the smallest number of parameters and calculations in the YOLOv7 series, and the convolution structure is highly plastic. YOLOv7 is a novel architecture in the YOLO series introduced by the official team of YOLOv4, including Chien-Yao Wang et al. in 2022 [18]. This experiment employs the lightweight version of YOLOv7, known as the YOLOv7-tiny algorithm. It retains the cascade-based model scaling strategy and enhances the efficient remote aggregation network [19] to ensure detection accuracy while requiring fewer parameters and achieving faster detection speed. The YOLOv7-tiny algorithm consists of three parts: the backbone network, neck network, and head network, as shown in excluding the red dashed area of Fig.3.

The feature fusion network of the neck network still adopts the Path Aggregation Feature Pyramid Network (PAFPN) from YOLOv5, integrating the high semantic information from the top layers of the Feature Pyramid Network (FPN) with the strong localization information tensors from the bottom-up Path Aggregation Network (PANet) [20]. This fusion of feature information achieves multiscale learning [20,21]. However, in the neck network of YOLOv7-tiny, the nearest-neighbor interpolation upsampling fails to integrate feature information from adjacent layers, leading to the loss of details and features such as information about small objects. Consequently, this reduction in the detection accuracy of mountain *T.Kirilowii* results in missed detections.

The head network model employed in this experiment utilizes the IDetect detection head [22]. It introduces an implicit representation strategy and refines prediction results by fusing feature values. However, the detection head of YOLOv7-tiny utilizes conventional convolutions, which may lead to the inability of the feature fusion in detection to focus accurately on the intended objects in cases of motion blur. This may result in the lack of objected strategies to enhance the detection performance of blurred objects generated during motion processes.

### 2.3 Improvement of the YOLOv7-tiny algorithm

**Fig 4.**
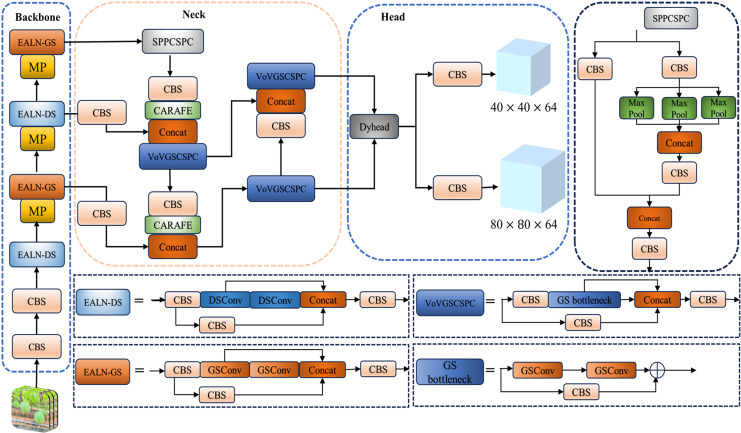
KPD-YOLOv7-GD network structure diagram.

To mitigate the limitations of the YOLOv7-tiny algorithm, this experiment introduces an enhanced variant, denoted as KPD-YOLOv7-GD, built upon the foundation of YOLOv7-tiny. The primary aim of this algorithmic refinement is to effectively cater to the requirements of mountain *T.Kirilowii* detection applications, thereby augmenting detection precision while concurrently minimizing computational complexity. The structure of the KPD-YOLOv7-GD algorithm is shown in Fig.4.

#### 2.3.1 Improvement of multi-scale feature layers.

**Fig 5.**
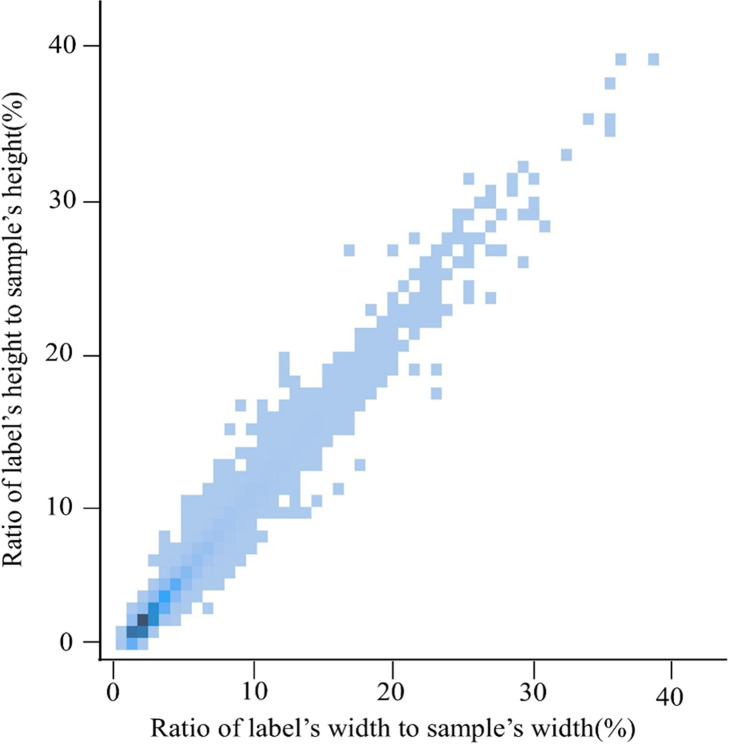
The width-to-height ratio of mountain *T.* ***Kirilowii* fruits within the sample images.** Therefore, this experiment proposes adding a small object detection layer to enhance the base algorithm. The specific implementation method is as follows: Firstly, within the existing detection layers, an additional detection head of size 160 × 160 is introduced to achieve a 4 × 4pixel receptive field, enabling the capture of finer details for smaller objects. Next, a set of feature layers composed of CBS convolutional modules, upsampling layers, Concat concatenation layers, and an ELAN-A layer is added to the original model, inserted between the 17th and 18th layers. Therefore, the original algorithm’s three-scale layers are elevated to four scales: 80 × 80, 40 × 40, 20 × 20, and the newly added 160 × 160, as shown in the red dashed area in Fig.3. Finally, an experiment is demonstrated to determine the most suitable detection scale for *T.Kirilowii*. The improvement reduces model parameters and computational burden while minimizing the loss of shallow information for small objects. Consequently, it enhanced the small objects of *T.Kirilowii* recognition under occlusion.

The mountain *T.Kirilowii* plants grow in clusters with dense foliage, making the fruits prone to being obscured. To distinguish mountain *T.Kirilowii* fruits from the background, collect its images and plot the distribution of width-to-height ratios of its dataset labels. As shown in Fig.5, the darker color indicates a higher quantity of mountain *T.Kirilowii* fruits within that range, suggesting that mountain *T.Kirilowii* fruits are primarily distributed within the width-to-height ratio range of [0.02, 0.3]. Hence, it can be observed that the proportion of width and height labels for mountain *T.Kirilowii* fruits is within 5%, indicating that mountain *T.Kirilowii* fruits are considered small object detection.

#### 2.3.2 Design of lightweight ELAM-A structure.

The regular convolutional layer requires many parameters and computational resources to achieve high accuracy, as its feature maps contain considerable redundant information. This experiment proposes replacing regular convolutional layers with DSConv [23] and GSConv [24] to reduce the model’s computational overhead. The specific improvement approach involves replacing the regular convolutional layers in the third and fourth layers of the ELAN-A backbone network with DSConv or GSConv.

**Fig 6.**
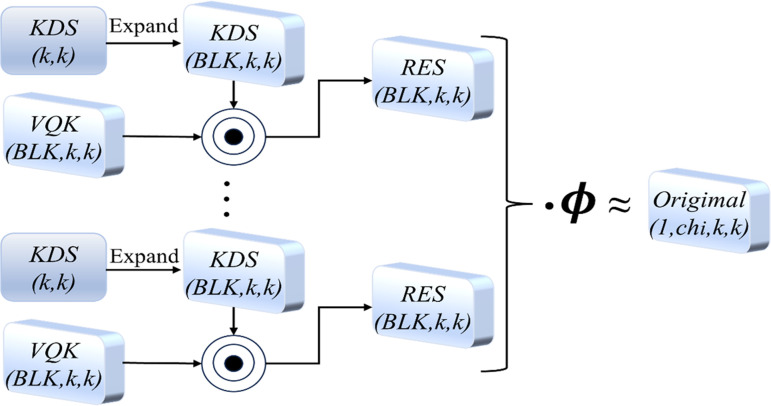
General idea of DSConv. The introduction of DSConv and GSConv reconstructed efficient remote aggregation network modules at different positions, which may result in variations in the model’s overall performance. Comparing the parameter count and computational complexity between regular convolution and GSConv reveals that GSConv can reduce redundant feature maps. When the computational complexity of the GSConv module is represented as *FLOPs1* and the conventional convolution module is represented as *FLOPs2,* the relationship between their computational complexities can be expressed as shown in Equation (1).


DSConv is divided into kernel Variable Quantized (VQK) and Distributed Shift (KDS). It achieves lower memory usage and higher speed in VQK by only storing integer values. Simultaneously, it maintains the same output as the original convolutional kernel through kernel-based and channel-based distributed shifting. as shown in Fig.6. For convenience and notation, let the original convolution tensor have size (*ch*_*o*_*, chi, k, k*), where *Ch*_*o*_ is the number of channels in the next layer, chi is the number of channels in the current layer, and *k* is the kernel’s width and height, where *BLK* is the block-size hyperparameter. Distributed offset convolution is a variant of Depthwise Separable Convolution (DSC) [25], known as the lightweight convolution. It retains the characteristic of DSC by increasing 1 to 1 dimension convolution on channels, utilizing convolutional operations to obtain the desired features while reducing network parameters. Moreover, it adds a learnable convolutional kernel to enhance the model’s performance further.

$$\frac{FLOPs1}{FLOPs2}=\frac{W\times H\times C_{1}\times K_{1}\times K_{2}\times C_{1}\times C_{2}\times 1\times 1}{W\times H\times C_{1}\times K_{1}\times K_{2}\times C_{2}}=\frac{1}{C_{1}}+\frac{1}{K_{1}\times K_{2}}=S$$ 1)

**Fig 7.**
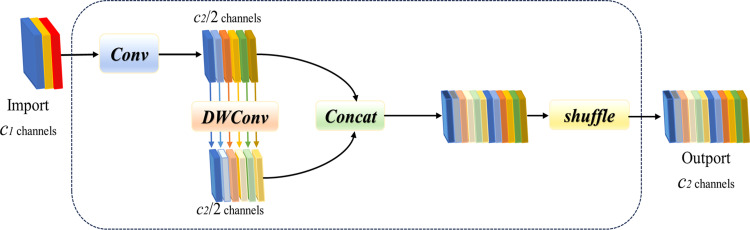
Structure of GSConv network.

Where *C*_*1*_ and *C*_*2*_ represent the number of channels in the input and output feature maps, respectively, *K*_*1*_ *×  K*_*2*_ denotes the kernel size of the convolution, and *S* represents the computational load, taking values between 0 and 1. *W* and *H* represent the width and height of the feature map, respectively. The parameters of each layer vary with the size of the convolutional kernel and the number of channels in the input feature maps. It can be observed that the parameter count of a regular convolution is *S* times that of a GSConv convolution. The GSConv structure is illustrated in Fig.7.

#### 2.3.3 CARAFE lightweight upsampling structure.

Feature upsampling plays a critical role in multiscale feature fusion for object detection frameworks, particularly within feature pyramid architectures. In the YOLOv7-tiny baseline, nearest-neighbor interpolation serves as the default upsampling operator due to its computational simplicity and hardware compatibility. However, this approach constructs fixed-weight convolutional kernels based exclusively on pixel spatial coordinates, inherently neglecting contextual feature semantics during the resolution enhancement process, thereby limiting its adaptability to complex feature interactions in cluttered detection scenarios. It boasts high computational efficiency and speed yet overlooks the insertion of new elements between pixel points, which can lead to the loss or distortion of image details. This experiment introduces CARAFE [26] to enlarge the information-receptive field of feature maps and enhance the semantic relevance of feature maps. It dynamically generates adaptive convolutional kernels, effectively aggregating contextual information. The CARAFE is a lightweight operator that introduces minimal parameters and computational overhead while providing a high processing speed. It consists of two main modules: the upsampling kernel prediction module and the feature reassembly module.

**Fig 8.**
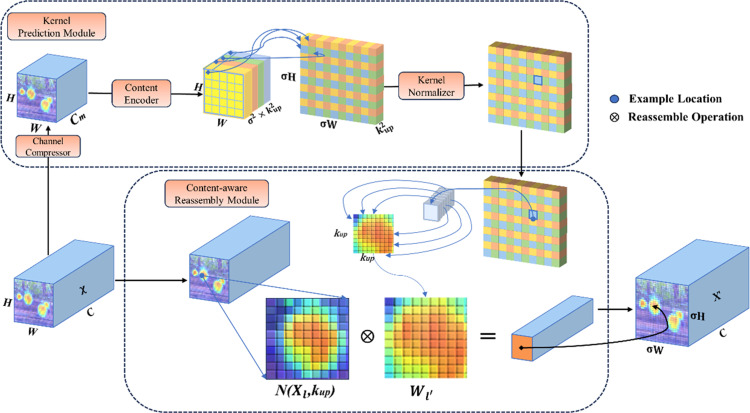
The overall framework of CARAFE.

When the upsampling factor is assumed to be *σ*, and the size of the input feature map is set as *C ×  H ×  W*, the specific steps of the upsampling kernel prediction module are as follows: First, compress the number of channels in the feature map to *C*_*m*_ using a *1 × 1* convolution, resulting in a compressed feature map of size *H ×  W ×  C*_*m*_. Channel compression of the feature map can effectively reduce the computation required in subsequent steps. Next, utilize a variable-sized *k*_*encoder*_ ×  *k*_*encoder*_ upsampling convolutional kernel to encode and predict the upsampling kernel, where larger kernels imply larger receptive fields and computational requirements. Furthermore, the input channels are assumed to be *C*_*m*_, and the output channels are set as *H × W × σ*^*2*^*k*^*2*^_*up*_. Due to the non-fixed nature of each position within the feature map, the corresponding feature convolution kernels are also non-fixed. Finally, the upsampling kernel is normalized using the Softmax function to ensure that the weights of the convolutional kernel sum up to 1. The overall framework of CARAFE is shown in Fig 8.

The content-aware reorganization module utilizes weighted summation operators to reconfigure features within local regions. The reorganization process is illustrated in Equation (2): each reorganized convolutional kernel *W*_*l’*_ mapping to the object position *l’* and the corresponding square region *N (X*_*l’*_*, k*_*up*_*)* with *l =  (i, j)* as its center.

Where $r=\left(k_{up}/2\right)$:


$$X_{l^{'}}=\sum_{n=-r}^{r}\sum_{m=-r}^{r}W_{l'}(n,m)\cdot X(i+n,j+m)$$ 2)

Compared to the nearest-neighbor interpolation sampling method, utilizing the CARAFE operator for feature map processing enables better focus on relevant point information within local regions, thereby capturing stronger semantic information. This characteristic endows the CARAFE operator with superior performance in tasks such as object detection.

#### 2.3.4 DyHead structure.

Due to the uneven fertility of mountainous terrain, the sizes of *T.Kirilowii* vary, resulting in significant differences during the picking and detection process. Hence, the network model’s detection head must possess the capability for dynamic real-time detection. Moreover, considering the motion blur interference caused by the robot’s movement during picking, the detection head requires a certain level of spatial awareness. This experiment introduces the DyHead algorithm in the detection head prediction stage to address the aforementioned issues. DyHead is a unified detection head method proposed by Dai et al. [27], which employs a multi-head attention mechanism. It utilizes attention mechanisms from three perspectives: scale perception, spatial position, and multitasking. In this way, the attention mechanism for the detection head is unified. In single-stage and two-stage object detection algorithms, DyHead can serve as a plug-and-play lightweight module. It can be conveniently embedded without increasing computational overhead, significantly enhancing the expressive power of the model’s detection head.

Before applying the DyHead detection head, a preparatory step involves generating a feature pyramid output $F_{in}={\left\{F_{in}\right\}}_{i}^{L}$ from the feature map of a backbone network. This process involves resizing features from different downsampling ratios to obtain three distinct scale feature layers. In the formula, L represents the number of layers in the feature pyramid. After concatenating different feature layers, a dimension tensor F ∈ RL ×  H ×  W ×  C is obtained, where H, W, and C, respectively, represent the height, width, and number of channels of the feature map. Here, denoted by S =  H ×  W, representing spatial position information, which can be expressed as ‘S’ instead of using ‘H’ and ‘W’ separately. The feature map can thus be viewed as a three-dimensional tensor with dimensions L, S, and C. After applying attention to a three-dimensional tensor, and these dimensions represent scale perception, spatial perception, and channel perception capabilities.

**Fig 9.**
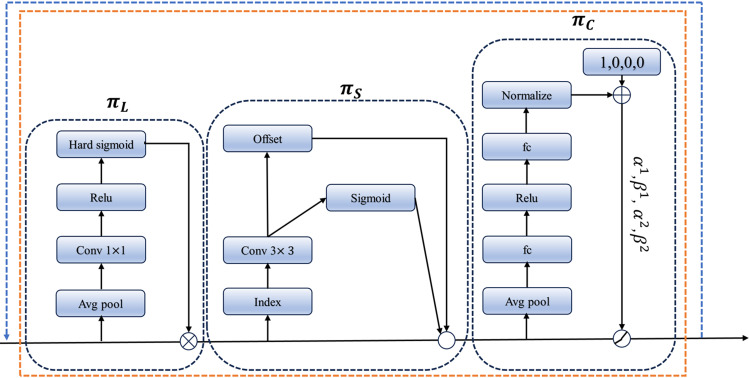
DyHead Structure.

The structural diagram of the DyHead Block is illustrated in Fig.9. The module is mainly composed of π_L_, π_S_, and π_C_ modules, connected in sequence, representing Scale-aware attention, Spatial attention, and Channel attention, respectively. After stacking these three attentions, a unified attention mechanism for the detection head is achieved. When the feature map is input into the π_L_ module, global average pooling is performed on S to obtain the maximum value of itself. Subsequently, a 1 × 1 convolution integrates all channels. Finally, the ReLU and hard sigmoid activation functions are utilized to extract distinctive features from the map. The process then moves to the π_S_ module, where a 3 × 3 convolutional layer from the deformable convolution v2 is utilized to modulate the input feature map, obtaining the offset of the feature map. In the π_C_ module, two fully connected neural network layers are employed for channel modeling, followed by global average pooling to reduce dimensionality and the number of parameters. Dynamic ReLU [28] is then applied to output different channel values based on the specific task.

#### 2.3.5 Channel pruning and knowledge distillation.

**Fig 10.**
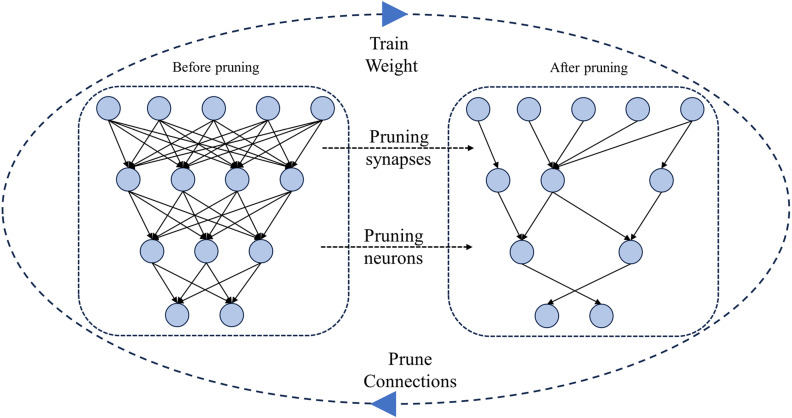
Channel pruning process. After channel pruning, the algorithm model experiences a significant reduction in parameters and computational load, yet it may incur a decrease in accuracy. To compensate for the decrease in accuracy after pruning, this experiment adopts knowledge distillation techniques to enhance the recognition accuracy of the model algorithm. Knowledge distillation is a model compression technique that enhances the performance and accuracy of a smaller model (student model) by transferring knowledge from a larger model (teacher model). Due to the strong generalization and robustness of the YOLOv7 model, it will be used as the teacher model in this experiment. KPD-YOLOv7-GD serves as the student model, as illustrated in Fig.11.

Channel pruning involves adding an L2 [29] regularization term to the loss function to constrain the weights. From an optimization perspective, the L2 regularization encourages sparsity in the weights, leading to many values in the weights being set to zero. This sparsity enables the removal of channels whose threshold values are below a specific coefficient. This process is repeated until the model achieves the desired size and accuracy. The principle is illustrated in Fig.10.

**Fig 11.**
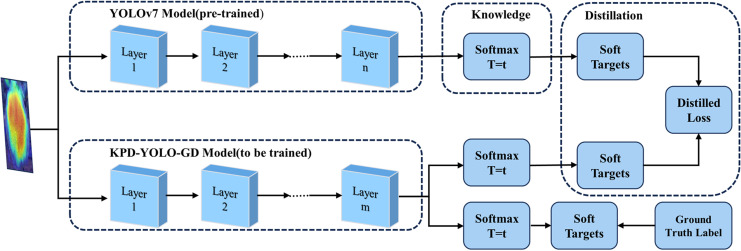
Improved model distillation structure.

The procedure incorporates the temperature parameter T into the Softmax function to achieve the distribution of soft objects. As shown in formula (3).


$$q_{i}=\frac{1}{\sum_{j}\exp(Z_{i}/T)}\exp(Z_{i}/T)$$ 3)

Where q_i_ represents the probability of category *i*, with values ranging from 0 to 1; Z_i_ represents the linear output input to the Softmax function; T represents the distillation temperature parameter.

## 3. Experimental design and results analysis

### 3.1 Experimental environment and parameter settings

The experiment was conducted in a Windows 11 operating system environment, utilizing PyCharm 2022 software to install the following environments: PyTorch 2.0.1, Python 3.10, and CUDA 11.8. The hardware configuration and experimental environment are detailed in Table 2.

**Table 2:** Hardware configuration and experimental environment.

Hardware	Configure	Environment	Version
systems	Windows11	Python	3.10.12
CPU	Core i9-13900HX	PyTorch	2.0.1
GPU	RTX4060	PyCharm	2022.3.3
RAM	8G	CUDA	11.8

During the training phase, excessive input image resolution may lead to issues such as insufficient memory and long. Therefore, the experiment set the image resolution to 640 pixels ×  640 pixels. The learning rate is a very important hyperparameter in deep learning, which controls the speed of updating model parameters during gradient descent optimization. The optimization function for this experimental model utilized stochastic gradient descent (SGD), setting the training total number of iterations to 200 epochs (training epoch), the initial learning rate to 0.01, the final learning rate to 0.01, the optimizer weight decay to 0.0005, and the momentum of SGD to 0.937 (SGD momentum). Batch size refers to the number of samples processed simultaneously in each round of gradient descent. A larger batch-size value can improve the training speed of the model. Therefore, the experiment set the batch size to 16. The main parameters of training are detailed in Table 3.

**Table 3:** The parameters of the training phase.

Parameter Name	Parameter Value
Image size	640 × 640
Batch size	16
Training epoch	200
Initial learning rate	0.01
Final learning rate	0.01
Optimizer weight decay	0.0005
Momentum	0.937

### 3.2 Evaluation indicators

In this experiment, a set of widely used evaluation indicators is employed to assess the performance of the KPD-YOLOv7-GD algorithm in the field of object detection. The evaluation indicators include precision (P), recall (R), mean Average Precision(mAP), and F1. The defined formulas are as follows:


$$P=\frac{TP}{TP+FP}$$ 4)

$$R=\frac{TP}{TP+FP}$$ 5)

Where TP is the number of correctly detected *T.Kirilowii* samples, FP is the number of incorrectly detected *T.Kirilowii* samples, and FN is the number of missed detections of *T.Kirilowii* samples.


$$AP=\int_{0}^{1}P(R)dR$$ 6)

$$mAP(0.5)=\frac{1}{n}\cdot \sum_{i=1}^{n}AP_{i}$$ 7)

$$F1=2\times \frac{P\times R}{P+R}$$ 8)

Where n is the number of categories, the mean Average Precision at loU threshold of 0.5(mAP0.5) is the average of the Average Precision (AP) values used to measure the overall average precision of the model. The mAP0.95 is the Average Precision calculated across different loU thresholds ranging from 0.5 to 0.95 with a step size of 0.05. The F1 score is the harmonic mean of P and R. In the experiment, higher accuracy in the F1 score and mAP indicates better model performance.

In terms of model complexity, a lightweight object detection algorithm proposed in this experiment emphasizes improvements to floating-point operations (FLOPs), parameter count (Parameters), and model size. As shown in formulas 9 to 11:


Parameters=[i×(c×c)×o]+o 9)

Where i, c, and o in the formulas are the input, convolution kernel, and output size, respectively.


$$FLOPs(Conv)=2\times H\times W\times (C_{imput}\times K^{2}+1)\times C_{out}$$ 10)

$$FLOPs(Pool)=\frac{W}{S}\times \frac{H}{S}\times C_{out}$$ 11)

Where W and H represent the width and height of the feature map, respectively. The variables H ×  W, C_input_, K, S, and C_out_ represent the output feature map size, input channels, kernel size, stride, and output channels, respectively. The total FLOPs of a model primarily originate from the sum of the FLOPs contributed by the convolutional layers and the average pooling layers within each layer of the model. While maintaining performance accuracy, reducing FLOPs and Parameters enhances the feasibility of deploying the improved algorithm model on small-scale mechanical devices.

### 3.3 Comparative experiments on detection multi-scale improvement

According to the analysis in section 2.3.1, the proportions of width and height labels for the *T.Kirilowii* fruits were both within 5%, indicating that *T.Kirilowii* fruits are small-scale objects. Therefore, in this experiment, a 160 × 160 detection scale is added to the baseline model, adjusting it to four detection scales: 180 × 80, 160 × 160, 40 × 40, and 20 × 20. To select the most suitable scale for detecting mountain *T.Kirilowii*, experiments are conducted based on two types of indicators: accuracy performance and complexity. The benchmark model YOLOv7-tiny scale is (80,40,20), while the comparison group detection scale combinations are (160,80,40,20), (160,80,40), (160,80), (40,20), and (80,40). The six different detection scale combinations are denoted as A1, A2, A3, A4, A5, and A6, respectively. The experimental results are shown in Table 4.

**Table 4:** Results of Combination Experiments at Various Detection Scales.

Modal	P(%)	R(%)	F1(%)	mAP0.5(%)	Parameters	FLOPs(G)	Size(MB)	FPS
A1	87.8	84	86	92.6	6014988	13.2	12.3	200
A2	87	82.5	85	91.4	6099616	15.3	12.7	196
A3	88.2	83.1	86	92.2	4057228	13.9	8.6	181.8
A4	89	83.3	86	92.6	3542888	12.2	7.5	198
A5	88.8	84	87	91.6	5977064	12.0	12.1	217.4
A6	87	85.9	87	92.8	3964680	11.4	8.2	212.8

As shown in Table 4, for scheme A4, the P score is 89%, with parameters and model size only being 3,542,888 and 7.5MB, respectively. Compared to scheme A1, the P has increased by 1.2%. Additionally, parameters and model sizes have been compressed by 40.10% and 39.02%. Importantly, scheme A4 demonstrates superior precision score, parameters, and model size performance compared to the other five schemes. This improvement can be attributed to introducing a larger 160 × 160 detection scale in scheme A4 while removing the 40 × 40 and 20 × 20 detection scales. The adjustment in scale strategy effectively enhances the accuracy of *T.Kirilowii* detection while reducing model complexity.

Regarding R, FLOPs, and frames per second (FPS), scheme A4 exhibits superior performance compared to schemes A2 and A3 but is lower than schemes A5 and A6. When comparing Scheme A6 to Scheme A4, improvements are observed in R, F1 score, and mAP0.5 by 2.6%, 1%, and 0.2%, respectively, alongside a reduction in FLOPs by 0.8G. This enhancement can be attributed to the strategic removal of the 20x20 detection scale in Scheme A6. This strategic adjustment effectively diminishes redundant parameters associated with detecting large objects, consequently reducing model complexity. Considering the overall accuracy, model complexity, and inference speed performance, scheme A6 outperforms scheme A4.

**Fig 12.**
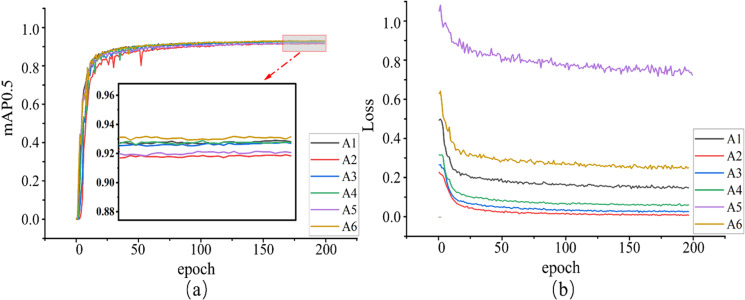
Training results of different detection scales (a) Comparison of the mean average precision values (b) Comparison of Loss values.

Overall, both scheme A4 and scheme A6 outperform the baseline model. Scheme A6 exhibits slightly better performance compared to scheme A4. It demonstrates the most comprehensive improvement. In terms of model accuracy, although the P score of Scheme A6 decreased by 0.8%, the R, F1 score, and mAP0.5 improved to 85.9%, 87%, and 92.8%, respectively, with an improvement of 1.9%, 1%, and 0.2%. Regarding model complexity, the parameters and FLOPs are reduced by 65.99% and 86.36%, respectively, compared to the baseline network. The model size is compressed from 12.3 MB to 8.2 MB, and the frame rate is increased from 200 to 212.8. The detection accuracy and robustness of the model algorithms with different detection scales can be reflected through the mAP0.5 and overall loss function obtained during model training. The training results are shown in Fig 12.

Fig. 12a and 12b show that the algorithms with six different detection scales converge around 100 epochs. Among them, schemes A2 and A4 exhibit more significant fluctuations in mAP0.5, while scheme A5 shows larger fluctuations in the loss function. These three schemes demonstrate poor robustness. Scheme A6 demonstrates the fastest convergence speed in both mAP0.5 and loss function. Additionally, it achieves high precision in mAP0.5. According to Table 3, scheme A3’s mAP0.5 decreases by 0.4% compared to the baseline model, indicating that it performs less optimally in lightweight compared to scheme A6. Based on a comprehensive evaluation considering model accuracy and complexity, this experiment has adopted scheme A6, namely YOLOv7-G.

### 3.4 Lightweight experiments

#### 3.4.1 Comparison of different lightweight.

This experiment introduces DSConv and GSConv into the backbone network of the improved YOLOv7-G architecture to select the optimal lightweight solution. Additionally, DSConv and the Slim-neck structure were incorporated into the neck network. Eleven experimental setups were designed for evaluation. Among these, schemes 1 to 4 were designed as single-module experiments, schemes 5 to 8 were designed as full-network dual-module hybrid experiments, and schemes 9 to 11 were designed as mixed-module experiments within the backbone network. The experimental results are presented in Table 5. In the table, “√” indicates the module’s usage, while “×” indicates that the module was not used.

**Table 5:** Result of comparison of different lightweight.

Method	Backbone		Neck		mAP0.5(%)	Parameters	FLOPs (G)	Size(MB)
	DSConv	GSConv	DSConv	Slim-neck				
1	**√**	**×**	**×**	**×**	92.9	3965640	7.6	14.6
2	**×**	**√**	**×**	**×**	92.7	3199176	9.7	6.6
3	**×**	**×**	**√**	**×**	92.5	3965000	10.7	8.9
4	**×**	**×**	**×**	**√**	93.1	3910664	10.9	8.1
5	**√**	**×**	**×**	**√**	92.9	3910664	7.2	14.5
6	**×**	**√**	**√**	**×**	92.8	3199176	9.0	7.3
7	**×**	**√**	**×**	**√**	92.6	3139304	9.1	6.6
8	**√**	**×**	**√**	**×**	92.7	3965960	6.9	15.3
9	**√**	**√**	**×**	**×**	92.8	3351848	8.7	8.2
10	**√**	**√**	**√**	**×**	92.7	3351848	8.0	8.9
11	**√**	**√**	**×**	**√**	92.9	3291976	8.0	8.1

As shown in Table 5, schemes 1 and 2 introduce DSConv and GSConv into the backbone network. Compared to scheme 2, scheme 1 improves mAP0.5 to 92.9%, with a 0.2% improvement. Scheme 1 achieves a 0.2% improvement in mAP0.5 compared to scheme 2. The parameters and model size have increased by 19.33% and 54.79%, respectively, while FLOPs have been compressed by 21.65%. scheme 3 introduces DSConv into the neck network, while scheme 4 introduces the Slim-neck structure. Compared to Scheme 3, Scheme 4 demonstrates an increase in mAP0.5 to 93.1%, representing a gain of 0.6%. Additionally, parameters and model size are compressed by 54336 and 0.8M, respectively, while FLOPs increase by 0.2G. In terms of lightweight performance, schemes 2 and 4 outperform schemes 1 and 3, while in terms of accuracy, scheme 2 slightly lags behind scheme 1, while scheme 4 outperforms scheme 3.

To further explore the balance between lightweight design and accuracy, schemes 5 to 8 are demonstrated. This scheme is primarily focused on introducing DSConv or GSConv into the backbone network and DSConv or Slim-neck into the neck network. In terms of accuracy, schemes 5 and 6 achieve higher precision than schemes 7 and 8. Among them, scheme 5 achieves the highest precision, reaching 92.9%. Scheme 7 achieves the lowest precision, reaching 92.6%, with only a 0.3% difference from scheme 5. Regarding lightweight design, schemes 5 and 6 demonstrate better lightweighting than schemes 7 and 8. Among them, scheme 7 achieves the best lightweight effect, with parameters and model size compressed to 3139304 and 6.6MB, respectively. In terms of balancing lightweight design and accuracy, schemes 5 and 7 need to retain the common Slim-neck design and can be further optimized. Therefore, Schemes 9 to 11 are designed.

Scheme 9 introduces DSConv and GSConv into the backbone network, while scheme 11 integrates schemes 5 and 7. Compared to scheme 9, scheme 11 improves mAP0.5 to 92.9%, with 0.1% improvement. Additionally, parameters, FLOPs, and model size are compressed by 59,872, 0.7G, and 0.1MB, respectively, ensuring both lightweight design and accuracy. Scheme 11 also outperforms 10 in terms of overall performance. Furthermore, through experiments with schemes 4, 7, and 11, it is observed that the Slim-neck lightweight structure has a minor impact on the model and even has a positive effect on improving the mAP0.5 value. This indicates that the Slim-neck module while maintaining a lightweight design, better captures image features, enhances the model’s representational capacity, and is more suitable for deployment on edge devices. Considering both model accuracy and complexity, the balanced scheme 11 is selected. It is named YOLOv7-GD.

#### 3.4.2 Channel pruning experiments.

The YOLOv7-G model demonstrates significant reductions in parameters, computational load, and model size through lightweight convolution techniques. However, it still faces challenges in its application to small-scale harvesting robots for mountain *T.Kirilowii*. Therefore, this experiment conducts channel pruning on the lightweight YOLOv7-GD algorithm to further reduce the complexity and size of the model. Six groups of trials with pruning rates ranging from 10% to 60% were designed to validate the impact of pruning at different levels on model performance. As shown in Table 6.

**Table 6:** Comparison of different pruning rates.

Pruning (%)	P (%)	R(%)	t (%)	mAP0.5(%)	Parameters	FLOPs(G)	Size(MB)
0	88.4	85.3	87	92.9	3291976	8.0	8.1
10	88.7	84.7	86	92.7	2512382	6.4	6.3
20	87.6	84.1	86	92	1484749	4.1	3.9
30	85.1	85.1	85	91.5	992739	3.0	2.7
40	84.8	82	84	89.9	472054	1.3	1.4
50	83.3	79.8	82	87.5	210916	0.6	0.8
60	82.3	79.8	81	87.4	126133	0.5	0.6

Compared to the unpruned model, pruning the model at rates of 10% and 20% resulted in a 1% decrease in F1 score and a respective decrease of 0.2% and 0.9% in mAP0.5. Regarding complexity, the number of parameters decreases by 23.68% and 54.90%, while FLOPs decrease by 20% and 48.75%, respectively. The model size is compressed by 22.22% and 51.85%, respectively. When pruning the model at rates of 30% and 60% compared to the unpruned model, there is a decrease in the F1 score of 2% and 6%, respectively, and a decrease in mAP0.5 of 1.4% and 5.5%, respectively. Regarding complexity, the number of parameters decreases by 69.84% and 96.16%, while FLOPs decrease by 62.50% and 93.75%, respectively. The model size is compressed by 66.67% and 92.59%, respectively.

The model with a 60% channel pruning rate exhibits the most optimal lightweight effect, with complexity and model size compressed by over 90%. However, the model’s mAP0.5 is only 87.4%, indicating a decrease of 5.5% compared to the baseline model. This makes it challenging to meet the accuracy requirements for mountain laurel detection. The decrease in mAP0.5 can be attributed to excessive pruning, which affects the integrity of the network structure by significantly reducing feature extraction parameters, resulting in a sharp decline in model accuracy. For models with channel pruning rates of 10%, 20%, and 30%, mAP0.5 decreased to 92.7%, 92%, and 91.5%, respectively, compared to the unpruned model. Specifically, it decreases sequentially by 0.2%, 0.9%, and 1.4%. The average compression of model complexity and size for these models is 21.97%, 51.84%, and 66.34%, respectively. Therefore, this experiment performs channel pruning on the lightweight YOLOv7-GD algorithm to further reduce the complexity and size of this model.

Considering the balance between lightweight design and accuracy, it’s evident that the model with a 20% pruning rate performs the best. It achieves a 51.84% optimization in lightweight design with only a 0.9% decrease in accuracy. Moreover, the significant reduction in model complexity does not compromise the integrity of the network structure. The experiment validates that appropriate pruning significantly enhances the lightweight nature of the model by reducing a considerable amount of redundant parameters in shallow layers. However, excessive pruning results in the model’s inability to learn sufficient information and features from the data, leading to a significant decrease in average precision for object detection.

**Fig 13.**
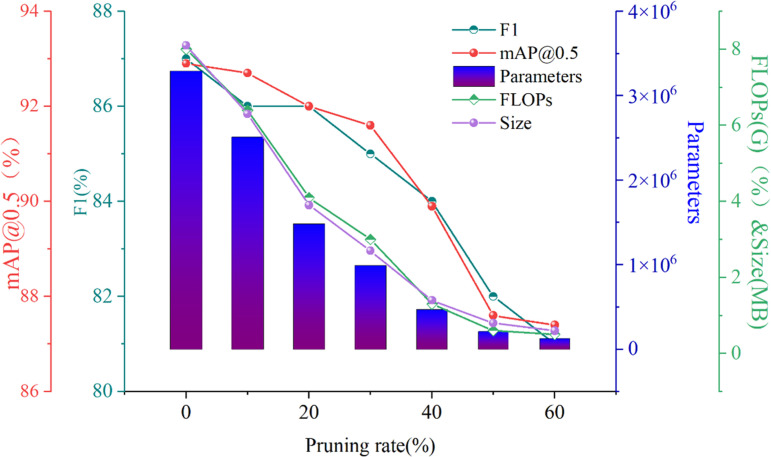
Comparative experiments with different pruning rates.

Overall, the model’s accuracy gradually decreases as the channel pruning rate increases, while its lightweight nature becomes more optimal with the increasing channel pruning rate. As shown in Fig.13, the accuracy performance indicators F1 score and mAP0.5 coincide at a pruning rate of 20%. Throughout the entire pruning process, this rate exhibits the smallest decrease in performance. Additionally, compared to a pruning rate of 10%, the model’s Parameters and FLOPs decreased by 40.90% and 35.94%, respectively. In the overall context, the most significant decrease in performance is observed, with the model size compressed by 38.10% compared to the baseline. This pruning rate also demonstrates the most optimal lightweight effect. Combining model accuracy and lightweight effectiveness, the solution with a 20% pruning rate is named P-YOLOv7-GD and was selected for comprehensive evaluation.

### 3.5 Experiments on Lightweight Accuracy Enhancement

Performance experiments were conducted to validate the effectiveness of DyHead and CARAFE lightweight upsampling structures in enhancing the detection accuracy of P-YOLOv7-GD. The introduction of the DyHead structure enhances the accuracy of the pruned algorithm, while the introduction of the CARAFE lightweight upsampling structure strengthens the algorithm’s robustness. The results are shown in Table 7.

**Table 7:** Comparison experiment of accuracy improvement.

Modal	mAP0.5(%).	mAP0.95(%)	Parameters	FLOPs(G)	Size(MB)
YOLOv7-tiny	92.6	58	6014988	13.2	12.3
P-YOLOv7-GD	92	56.7	1484749	4.1	3.9
P-YOLOv7-GD^+^Dyhead	92.3	57.4	1653349	4.2	4.2
P-YOLOv7-GD+CARAFE	92.1	56.9	1609309	4.4	4.1
P-YOLOv7-GD ^+^ DyHead ^+^ CARAFE	92.5	57.7	1777909	4.5	4.5

The improved algorithm P-YOLOv7-GD for mountain *T.Kirilowii* object detection undergoes lightweight operations to reduce the model’s computational costs. However, the performance of the model is also affected to some extent. Therefore, Dyhead and CARAFE modules are introduced to enhance P-YOLOv7-GD model accuracy and performance. Compared to the P-YOLOv7-GD model, the P-YOLOv7-GD+DyHead model improves mAP0.5 and mAP0.95 by 0.3% and 0.7%, respectively. The P-YOLOv7-GD+CARAFE model increases mAP0.95 by 0.2%. However, when both the DyHead and CARAFE modules are introduced to the P-YOLOv7-GD model simultaneously, compared to the P-YOLOv7-GD model, mAP0.5 and mAP0.95 improve by 0.5% and 1%, respectively. This is mainly attributed to the fact that CARAFE increases the information sensing field of feature maps and enhances the semantic information relevance of feature maps. This effectively aggregates contextual information, enhances the robustness of the model, and strengthens the ability of the DyHead detector to detect the semantic information of feature maps. Therefore, the simultaneous use of the two modules brings about a significant enhancement in model performance.

**Fig 14.**
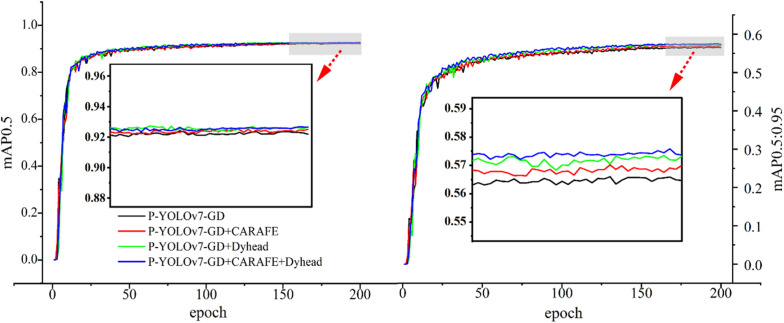
Comparison experiment of accuracy improvement.

As shown in Fig.14, without the introduction of CARAFE, the P-YOLOv7-GD algorithm exhibits large amplitudes in the trained mAP0.5 and mAP0.95, indicating poor robustness. The introduction of CARAFE and DyHead detection heads has improved accuracy and enhanced algorithm robustness. This lightweight algorithm, now with enhanced accuracy, is named PD-YOLOv7-GD.

### 3.6 Knowledge Distillation Experiments

In terms of model complexity, compared to the original baseline model, PD-YOLOv7-GD achieves reductions of 70.44% in Parameters, 65.90% in FLOPs, and 63.41% in model size. However, there is only a slight decrease of 0.1% in mAP0.5 and 0.3% in mAP0.95. To further enhance the model’s accuracy, this experiment adopted knowledge distillation to improve the performance of the PD-YOLOv7-GD algorithm. YOLOv7 is the teacher network, while the enhanced PD-YOLOv7-GD acts as the student network. A comparison was conducted with the original baseline model YOLOv7-tiny network. Four distinct distillation temperature experiments were designed for comparison. As shown in Table 8, the first column labeled “K” indicates the use of knowledge distillation on the PD-YOLOv7-GD model.

**Table 8:** Comparison of different distillation temperatures.

Modal	T	F1(%)	mAP0.5(%).	mAP0.95(%)
YOLOv7-tiny	0	86	92.6	58
PD-YOLOv7-GD	0	86	92.5	57.7
KPD-YOLOv7-GD	10	87	93	58.8
KPD-YOLOv7-GD	20	87	93.2	59
KPD-YOLOv7-GD	30	87	93.1	58.9
KPD-YOLOv7-GD	40	87	93	58.8

Compared to the model with a distillation temperature of 0, models with distillation temperatures of 10, 20, 30, and 40 all show improvements in F1 score by 1%, mAP0.5 by 0.5%, 0.7%, 0.6%, and 0.5%, and mAP0.95 by 1.1%, 1.3%, 1.2%, and 1.1%, respectively. The training mAP0.5 and mAP0.95 initially increase and then decrease with the rise of the distillation temperature T. The accuracy reaches its peak when the distillation temperature T is 20 degrees. Therefore, selecting an appropriate distillation temperature is crucial for the learning process of the student network. Through experimental comparison, a distillation temperature of T = 20 was ultimately chosen. Compared to the baseline model YOLOv7-tiny network, F1 score, mAP0.5, and mAP0.95 are improved by 1%, 0.6%, and 1%, respectively, demonstrating a significant enhancement in accuracy through knowledge distillation.

### 3.7 Ablation experiments

Four networks and the original network were selected for ablation experiments to validate the performance of the KPD-YOLOv7-GD model in mountain *T.Kirilowii* harvesting detection. As shown in Table 9, scheme 1 involves adjusting the detection scale of the original model. scheme 2 entails lightweight operations. scheme 3 introduces the Dyhead. scheme 4 introduces the CARAFE structure and performs knowledge distillation. The symbols “√” and “×” represent the adoption or non-adoption of the corresponding strategy in applying the model. The results of the ablation experiments are shown in Table 9.

**Table 9:** Comparison of experimental results at ablation.

Modal	Scaling strategy	Lightweight	Dynamic Head	CARAFE	mAP0.5 (%)	Parameters	FLOPs (G)	Size (MB)
YOLOv7-tiny	**×**	**×**	**×**	**×**	92.6	6007596	13.2	12.3
1	**√**	**×**	**×**	**×**	92.8	3964680	11.4	8.2
2	**√**	**√**	**×**	**×**	92	1484749	4.1	3.9
3	**√**	**√**	**√**	**×**	92.3	1653349	4.2	4.2
4	**√**	**√**	**√**	**√**	93.2	1777909	4.5	4.5

As shown in Table 9, after the lightweight improvement of the baseline network model, the complexity of the model is significantly reduced compared to the original network model. For scheme 1, Parameters are reduced to 65.99% of the baseline model, while FLOPs and model size are compressed from 13.2G and 12.3MB to 11.4G and 8.2MB, respectively. Additionally, mAP0.5 is improved by 0.2%. After conducting lightweight operations in scheme 1, scheme 2 achieves further reductions in Parameters, FLOPs, and model size by 62.55%, 64.03%, and 52.44%, respectively, compared to scheme 1. This further reduces the model complexity, with mAP0.5 only decreasing by 0.8%. With the introduction of the Dyhead, scheme 3 achieves a mAP0.5 that is 0.3% higher than Scheme 2, thus compensating for the accuracy loss caused by Scheme 2. After introducing the CARAFE structure and performing knowledge distillation, scheme 4 achieves a mAP0.5 of 0.9% higher than scheme 3, further enhancing the model’s accuracy. Additionally, schemes 3 and 4 have slightly higher model complexity than schemes 2. However, scheme 4 achieves a 1.2% improvement in accuracy compared to scheme 2. The final improved model is named KPD-YOLOv7-GD.

### 3.8 Comparative Experiments of Mainstream Single-Stage Lightweight Object Detection Algorithms

To further demonstrate the performance superiority of the KPD-YOLOv7-GD model, the performance of mainstream single-stage lightweight object detection algorithms was demonstrated. The algorithms considered including YOLOv3-tiny [30], YOLOv5s [31], YOLOv6s [32], YOLOv7-tiny and YOLOv8n [33]. This experiment is conducted under the same conditions. The results are shown in Table 10.

**Table 10:** Experimental comparison of mainstream lightweight detection algorithms.

Modal	mAP0.5 (%)	mAP0.95 (%)	Parameters	FLOPs(G)	Size (MB)	Speed (ms)
YOLOv3-tiny	88.4	55.8	12132642	19	24.4	5.7
YOLOv5s	92.6	58.8	7063542	16.5	14.4	3.8
YOLOv6s	90.2	56.6	17190000	44.1	40.6	8.5
YOLOv7-tiny	92.6	58	6007596	13.2	12.3	5
YOLOv8n	93	61	3011043	8.2	6.2	2.9
KPD-YOLOv7-GD	93.2	59	1777909	4.5	4.5	5.4

**Fig 15.**
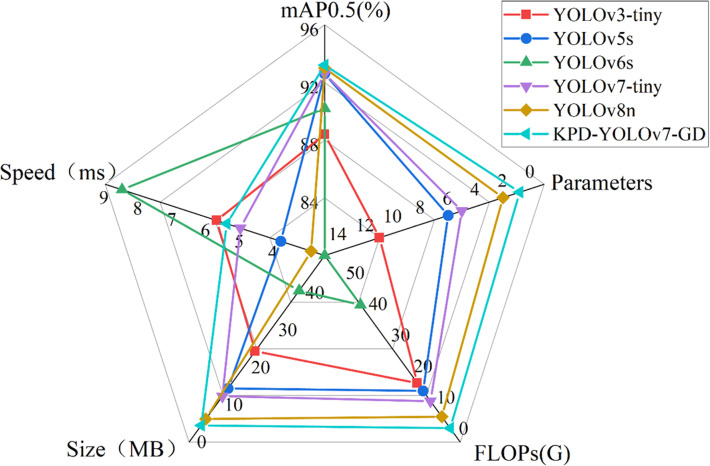
Performance comparison of mainstream lightweight object detection algorithms.

Table 10 shows that on the self-built mountain *T.Kirilowii* dataset, the proposed KPD-YOLOv7-GD outperforms mainstream single-stage lightweight object detection algorithms in terms of average detection accuracy. The model size is also compressed by 85.34%, 68.75%, 88.91%, 63.41%, and 27.42%, respectively. Parameters are reduced by 85.35%, 74.83%, 89.66%, 70.41%, and 40.95%, respectively. FLOPs decreased by 76.31%, 72.73%, 89.80%, 65.90%, and 45.12%, respectively. The model performs well in terms of accuracy, except for mAP0.95 being lower than YOLOv8n. In terms of detection speed, compared to YOLOv5s, YOLOv7-tiny, and YOLOv8n models, the inference detection speed of our model increases by 1.2ms, 0.4ms, and 2.1ms, respectively. However, it still meets the real-time detection requirements for mountain *T.Kirilowii*. The visualization results of mainstream single-stage lightweight object detection algorithms are shown in Fig.15.

As shown in the radar chart (Fig.15), the cyan line corresponds to the model proposed in this experiment, KPD-YOLOv7-GD. Compared to other mainstream lightweight object detection algorithms, KPD-YOLOv7-GD shows slightly lower inference detection speed compared to the YOLOv5s, YOLOv7-tiny, and YOLOv8n models. However, KPD-YOLOv7-GD significantly reduces model size, Parameters, and FLOPs. Additionally, there is a slight improvement in the mAP0.5 value. The experimental results indicate that KPD-YOLOv7-GD outperforms other mainstream lightweight object detection algorithms in terms of both accuracy performance and complexity. Thus, it can better meet the requirements for lightweight applications and high precision recognition in mountain *T.Kirilowii* small-scale harvesting robots.

## 4. Discussion

### 4.1 Visualization analysis

**Fig 16.**
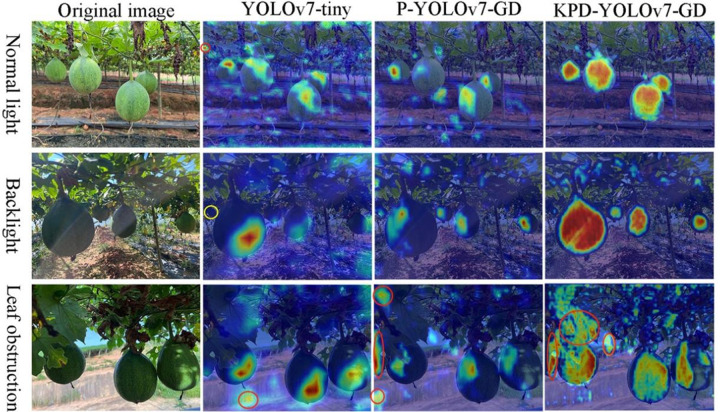
Comparative visualization of feature maps optimized by algorithms at different levels.

This experiment started with the YOLOv7-tiny base model and incrementally optimized it. Beyond section 3.5, the enhanced network KPD-YOLOv7-GD began to outperform the baseline network in terms of performance. In particular, the mAP0.5 value of KPD-YOLOv7-GD reaches 93.2%, surpassing the lightweight improved network P-YOLOv7-GD by 1.2% and the baseline network YOLOv7-tiny by 0.6%. To provide a more intuitive comparison of the algorithm’s effectiveness in mountainous environments for mountain *T.Kirilowii* detection during the improvement process, gradient-weighted class activation mapping (Grad-CAM) heatmaps [34] were used as a visualization analysis tool. Grad-CAM was a gradient-based method used to generate class activation maps. It allocates importance to individual neurons based on the gradient information flowing into the final convolutional layer of a CNN. This approach allows for better visualization of features during the improvement process of the algorithm. Images involving complex backgrounds with ‘normal light,’ ‘backlight,’ and ‘obstruction’ were selected, and Grad-CAM was applied to them. As shown in Fig 16, the regions colored in red signify a higher contribution to detection accuracy, whereas the blue-colored regions indicate a lower contribution.

In Fig.16, the first column represents the original image, while the others depict the feature heatmap visualizations of YOLOv7-tiny, P-YOLOv7-GD, and KPD-YOLOv7-GD, respectively. The red circles indicate false positive results for *T.Kirilowii* detection, while the yellow circles denote false negative results. For KPD-YOLOv7-GD, the red areas are uniformly centered and distributed evenly on the *T.Kirilowii*, indicating a good recognition performance. Meanwhile, for the others, the red areas are not uniformly distributed. In YOLOv7-tiny, the heatmap recognition area is biased towards the lower right area of the fruit. In P-YOLOv7-GD, the heatmap recognition area is biased towards the upper left area of the fruit.

In normal light and backlight conditions, YOLOv7-tiny’s feature heatmap showed false positive results and missed a small object under backlighting, the mountain *T.Kirilowii*. However, both P-YOLOv7-GD and KPD-YOLOv7-GD show detection capability for small objects. In the KPD-YOLOv7-GD feature heatmap, the distribution of the red area is prominently centered on the mountain *T.Kirilowii*, effectively eliminating external background information and enhancing the focus on mountain *T.Kirilowii* detection.

All three algorithms exhibit varying degrees of false positive detection results in backlight and obstruction conditions. YOLOv7-tiny primarily generates false positives in detecting mountainous backgrounds, whereas P-YOLOv7-GD and KPD-YOLOv7-GD mainly produce false positives in detecting obstructed fruit foliage. However, the distribution effectiveness of the red area in KPD-YOLOv7-GD is superior to that of P-YOLOv7-GD. This is primarily attributed to the KPD-YOLOv7-GD algorithm’s incorporation of CARAFE and Dyhead, which enhance the contextual information and focus more on identifying mountain *T.Kirilowii*.

Therefore, the KPD-YOLOv7-GD model demonstrates superior performance in complex mountainous backgrounds, enhancing detection accuracy in mountain *T.Kirilowii* fruit detection. This provides a practical and effective solution for lightweight applications of mountain *T.Kirilowii* harvesting in complex mountainous environments. the experimental results thoroughly demonstrate the effectiveness of the KPD-YOLOv7-GD model.

### 4.2 Comparative analysis of recognition performance among similar algorithms

In similar studies, Zhang et al. [13] achieved the detection of apples using the YOLOv4 model. They employed the lightweight feature extraction network GhostNet as the backbone network of the model. Moreover, they introduced depth-wise separable convolutions in the neck and head of the model to further reduce the number of model parameters and decrease model complexity. This optimization results in an average precision of 93.83% and a recognition time of 23.75ms. Compared to traditional computer vision methods, this approach can identify the entire apple, significantly improving recognition speed and meeting the requirements for real-time detection. Nevertheless, the early YOLOv4 model still has room for improvement in terms of accuracy, and its size remains relatively large. Zeng [35] employed two methods to achieve lightweight improvements on YOLOv5s. One involved enhancing YOLOv5s’ neck using the lightweight network MobileNetV3, while the other utilized network refinement algorithms to perform channel pruning on the tomato recognition model within YOLOv5s. After applying both improvement methods, the mAP0.5 reached 95.5%, a decrease of 1.9% compared to the baseline model. Regarding model size, parameters, and FLOPs, both methods resulted in reductions of 23.61%, 22.01%, and 17.07%, respectively, compared to the baseline model. Indeed, this academic endeavor laid the foundation for a comprehensive dataset comprising images of tomatoes captured under greenhouse conditions. The experiment effectively addressed the issue of tomato recognition in greenhouse environments while achieving model lightweight. However, it is worth noting that despite the efficiency advantages of lightweight models, there was indeed a slight decrease in detection accuracy compared to the baseline model.

Ou et al. [36] improved YOLOv7 by introducing ShuffleOne as a novel backbone network and slim-neck as a neck network. These enhancements led to a mAP0.5 of 90.45%. Furthermore, they compressed the model size to 36.62% of the original model and reduced the parameters to 37.26%. Compared to the baseline model, the methods described in this paper significantly increased mAP0.5 by 4.85% and reduced recognition time to 6.5ms. They also considered the complex situations during mountain passion fruit detection, such as lighting changes and leaf obstruction. Compared to the earlier experiment, KPD-YOLOv7-GD achieved a mAP0.5 of 93.2%, 0.6% higher than the baseline model. The model size, parameters, and FLOPs were also reduced to 36.59%, 29.59%, and 34.1% of the baseline model, respectively, with a recognition time of 5.3ms.

### 4.3 Limitations

The proposed method in this experiment focuses on object detection of mountain *T.Kirilowii* cultivation. It is influenced by the terrain and lighting conditions of the natural environment, leading to diverse perspectives for fruit detection, which poses challenges to the detection process. However, large-scale mechanical harvesting in mountainous environments is not feasible, highlighting the clear advantages of localized applications of small-scale harvesting robots. Nonetheless, small-scale harvesting robots are constrained by hardware resources and other factors, posing bottlenecks to devising miniaturization and hindering the ability to process object detection in real time effectively. To address these challenges, GSConv and model channel pruning techniques reduce model complexity, ensuring the practical feasibility of model deployment on small-scale devices. Applying the KPD-YOLOv7-GD model to small-scale harvesting robots and other agricultural equipment holds significant importance for the intelligence of mountain agriculture and the utilization rate of mountainous regions. Compared to controlled environments like greenhouse agriculture and traditional field farming methods, mountainous cultivation faces even more daunting challenges due to its varied terrain and diverse lighting conditions, which impact the accurate recognition of mountain *T.Kirilowii* by models. To address these challenges, dynamic modules for aggregating contextual information and model knowledge distillation techniques are employed to optimize the model, ensuring high efficiency and accuracy when dealing with large, complex mountainous backgrounds. Although this experiment has made some progress, several issues still need improvement. Future research should focus on optimizing image processing techniques to reduce the impact of lighting variations under different perspectives in mountainous detection. Additionally, proposing methods combining dark channel enhancement to address the issue of low-light images captured at night can enhance the accuracy and robustness of the model. Furthermore, factors such as device motion, vibration, and noise are important considerations affecting the detection accuracy and stability of the model. Accelerating the inference detection speed also requires further adjustments and optimizations of the model algorithm.

In summary, the KPD-YOLOv7-GD model holds significant agricultural application value. This method demonstrates excellent performance in lightweight model deployment possibilities and object detection accuracy, and it is capable of identifying and detecting under various lighting conditions during the daytime. Integrating it into small-scale agricultural equipment for mountainous regions holds promise for achieving intelligent harvesting in mountain agriculture. It offers a robust solution for small-scale harvesting in complex mountainous environments.

## 5. Conclusion

Detecting *T.Kirilowii* in complex mountain environments is crucial for its mechanized picking. Due to the environmental characteristics of brightness variation, high planting density, mutual occlusion, and motion blur occurring during the harvesting process, detection algorithms face problems with many parameters and high computational complexity. This experiment proposes a novel recognition method based on an improved YOLOv7-tiny framework, KPD-YOLOv7-GD. Firstly, a lightweight network structure is obtained by reducing the number of detection layers and restructuring the backbone network with the ELAN structure. Building upon this foundation, channel pruning is applied to the model to reduce model complexity further. Finally, CARAFE and Dyhead are introduced, and knowledge distillation is employed to compensate for accuracy loss. KPD-YOLOv7-GD achieved an F1 score of 87%, mAP0.5 of 93.2%, and mAP0.95 of 59%. Additionally, it had 1.78 million parameters, 4.5 billion FLOPs, and a model size of 4.5MB. The main contributions of this experiment are as follows:

(1) Adjusting the number of detection layers reduces the model’s complexity, improving performance. The experimental results demonstrate that the indicators R, F1 score, mAP0.5 improved by 1.9%, 1%, and 0.2%, respectively, through this method. Moreover, the parameters, FLOPs, and model size are compressed to 65.99%, 86.36%, and 66.67% of the original baseline network, respectively. This lays the foundation for high-precision and reliable model compression.
(2) By introducing DSConv, GSConv, and Slim-neck modules and conducting channel pruning, the complexity of the model is further reduced. Parameters, FLOPs, and model size were compressed by 75.29%, 68.94%, and 68.29%, respectively, compared to the baseline model. Meanwhile, mAP0.5 only decreased by 0.6%.
(3) Detection accuracy improved by incorporating CARAFE and Dyhead and conducting knowledge distillation. Compared to the baseline model, the mAP0.5 and mAP0.95 reached 93.2% and 59%, respectively, an improvement of 0.6% and 1%. This compensates for the accuracy loss caused by lightweight improvements and channel pruning.
(4) Comparing KPD-YOLOv7-GD with different mainstream single-stage lightweight detection algorithms such as YOLOv3-tiny, YOLOv5s, YOLOv6s, YOLOv7-tiny, and YOLOv8n, the mAP0.5 of the KPD-YOLOv7-GD model increased by 4.8%, 0.6%, 3%, 0.6%, and 0.2%, respectively. In terms of complexity, the parameters decreased by 85.35%, 74.83%, 89.66%, 70.41%, and 40.95%, while FLOPs decreased by 76.31%, 72.73%, 89.80%, 65.90%, and 45.12%, respectively. The model size was also compressed by 85.34%, 68.75%, 88.91%, 63.41%, and 27.42%, respectively.

The experimental results demonstrate that the KPD-YOLOv7-GD model algorithm outperforms similar algorithms. It not only meets the lightweight requirements for mountain *T.Kirilowii* recognition but also achieves higher detection accuracy and lower complexity, increasing the model’s potential for lightweight application. The proposed method provides an effective object detection approach for mountain *T.Kirilowii* fruit harvesting in mountainous agriculture, offering valuable research insights for small-scale equipment in mountain agriculture.
